# User fee policies and women’s empowerment: a systematic scoping review

**DOI:** 10.1186/s12913-020-05835-w

**Published:** 2020-10-27

**Authors:** Veronique Plouffe, Frank Bicaba, Abel Bicaba, Thomas Druetz

**Affiliations:** 1 Independent Consultant, Montreal, Canada; 2 Société d’Études et de Recherches en Santé Publique (SERSAP), Ouagadougou, Burkina Faso; 3 University of Montreal School of Public Health, Montreal, Canada; 4 Centre de Recherche en Santé Publique, Montreal, Canada; 5 Center for Applied Malaria Research and Evaluation, Tulane University, New Orleans, USA

**Keywords:** Women’s empowerment, women’s agency, User fee policies, Access to healthcare, Systematic review, Low- and middle-income countries

## Abstract

**Background:**

Over the past decade, an increasing number of low- and middle-income countries have reduced or removed user fees for pregnant women and/or children under five as a strategy to achieve universal health coverage. Despite the large number of studies (including meta-analyses and systematic reviews) that have shown this strategy’s positive effects impact on health-related indicators, the repercussions on women’s empowerment or gender equality has been overlooked in the literature. The aim of this study is to systematically review the evidence on the association between user fee policies in low- and middle-income countries and women’s empowerment.

**Methods:**

A systematic scoping review was conducted. Two reviewers conducted the database search in six health-focused databases (Pubmed, CAB Abstracts, Embase, Medline, Global Health, EBM Reviews) using English key words. The database search was conducted on February 20, 2020, with no publication date limitation. Qualitative analysis of the included articles was conducted using a thematic analysis approach. The material was organized based on the Gender at Work analytical framework.

**Results:**

Out of the 206 initial records, nine articles were included in the review. The study settings include three low-income countries (Burkina Faso, Mali, Sierra Leone) and two lower-middle countries (Kenya, India). Four of them examine a direct association between user fee policies and women’s empowerment, while the others address this issue indirectly —mostly by examining gender equality or women’s decision-making in the context of free healthcare. The evidence suggests that user fee removal contributes to improving women’s capability to make health decisions through different mechanisms, but that the impact is limited. In the context of free healthcare, women’s healthcare decision-making power remains undermined because of social norms that are prevalent in the household, the community and the healthcare centers. In addition, women continue to endure limited access to and control over resources (mainly education, information and economic resources).

**Conclusion:**

User fee removal policies alone are not enough to improve women’s healthcare decision-making power. Comprehensive and multi-sectoral approaches are needed to bring sustainable change regarding women’s empowerment. A focus on “gender equitable access to healthcare” is needed to reconcile women’s empowerment and the efforts to achieve universal health coverage.

## Background

Achieving gender equality and empowering women and girls, as well as achieving universal health coverage (UHC) are part of the 2030 Agenda for Sustainable Development [1]. Over the past decade, there has been growing international recognition that health, gender equality, women’s empowerment and sustainable development are intricately related [2]. While the United Nations Millennium Development Goals (MDGs) commitments to gender equality and women’s empowerment (MDG #3) focused on gender parity in education and reducing maternal mortality (MDG #5), the Sustainable Development Goals (SDGs) address gender equality not only as a stand-alone goal (SDG 5), but as a cross-cutting development issue [1, 3]. In 2030 Agenda, gender equality is now considered essential to achieve other goals, including those related to poverty, nutrition, employment and health [2]. Gender inequalities have been recognized as powerful determinants of health and well-being and, as such, should be targeted in the efforts to increase access to healthcare. This problem is particularly salient in low- and middle-income countries (LMICs), where barriers to healthcare access are prioritized for intervention on the road to Universal Health Coverage (UHC) [4, 5].

User fee reduction or abolition policies are among the most prominent strategies to achieve UHC in LMICs [6–8]. Several systematic reviews have shown their effectiveness in increasing access to preventive and curative healthcare services among the targeted population, generally children under 5 years of age and pregnant women [9–15]. Removing user fees also changes treatment-seeking practices by decreasing self-medication and traditional treatments, by increasing the number of visits to health centers and decreasing delay in seeking treatment [16]. Evidence suggests that these policies are associated with a reduction in neonatal, maternal and child mortality [17–20]. For these reasons, a growing number of LMICs have partially or completely removed user fees for pregnant women and/or for children under five. According to the World Bank, 46 of the 54 African countries have taken measures to that end since the turn of the Millennium [21]. This is a major reorientation of health policies. User fee abolition initiatives overturn the cost recovery system which imposed direct payment for healthcare in most sub-Saharan African (SSA) countries following the Bamako Initiative in 1986 [22].

Despite the large number of studies that have addressed user fee policies, their repercussions on women’s empowerment or gender equality has never been systematically reviewed. This is surprising, especially considering the numerous reviews that have identified the relationship between women’s empowerment and other types of finance-based interventions (i.e., cash transfer, micro-credit, self-help groups, etc.) [23–26]. While some studies indicate that user fee abolition can decrease health inequities, most have focused on spatial or economic disparities [20, 27, 28]. How elimination of fees can affect gender-based inequities and women’s empowerment related to healthcare remains to be investigated. In recent years, there has been growing commitment towards women’s empowerment, for its intrinsic value (achieving their rights and well-being), but also valued for its positive spillover effects – leading to improved outcomes in poverty reduction, health and education [29]. While acknowledging its polysemic nature, for the purpose of this paper, we adhere to Kabeer’s definition of empowerment (2005), which refers to the “processes by which those who have been denied the ability to make choices acquire such an ability” and which is constituted of three interrelated dimensions: agency, resources and achievements.

This literature review was conducted to fill this important knowledge gap. Its aim is to systematically review the evidence on the repercussions of user fee policies in LMICs on women’s empowerment, including but not limited to their autonomy in decision-making related to healthcare. Using a gender lens, the scope of this review is to address the following questions:

I. Does user fee removal, reduction, or introduction influence women’s empowerment in LMICs?
II. What are the repercussions of these user fee policies on women’s autonomy in health-related decision-making in LMICs?

Finally, the secondary objective of this review is to identify and qualitatively assess the usefulness of a theoretical framework suitable for gender-focused literature reviews. These findings are intended to inform future research and promote women’s empowerment as a prism for planning and assessing universal health coverage policies.

## Methods

This systematic scoping review (SSR) uses the methodological framework developed by Peters et al. [30] Like scoping reviews, SSRs consider a broad research question with the aim of mapping literature and synthesizing key evidence on a topic gathered from different disciplines and with different study designs. This approach supports our objective, which is to clarify the relationship between user fee policies, reproductive healthcare and women’s empowerment. Like systematic reviews, SSRs use standardized procedures. Notably, SSRs follow the same methodology as systematic reviews for searching published and unpublished literature, defining inclusion and exclusion criteria, and describing all methodological steps in order to allow the review to be replicated. This review follows the Preferred Reporting Items for Systematic Reviews and Meta-Analyses (PRISMA) guidelines.

### Search strategy

Following the guidance for SSR, a three-step approach was used [30]. The first step consisted in identifying the keywords that refer to the research topic. A limited search was performed in two databases (Medline and Google Scholar) to list the various terms used in the literature to mention this review’s two main concepts: “women’s empowerment” and “free healthcare” (for a more detailed definition of women’s empowerment, see Table 1). There was no restriction on type of publication, study design, or methods used. The list of terms was then compared to those used in other systematic reviews on user fee removal. Finally, the list was discussed with team members for additional suggestions. The search was not performed by referring to the location or venue type where healthcare is provided (such as: maternity clinics, dispensaries, hospitals, health facilities, etc.).

**Table 1:** Definition of Women’s Empowerment

Women’s empowerment
Using Kabeer’s definition, empowerment refers to the “processes by which those who have been denied the ability to make choices acquire such an ability”. It is constituted of three interrelated dimensions:
(i) Agency, i.e. the processes by which choices are made and put into effect. In relation to empowerment, agency implies actively exercising choice and doing so in ways that challenge power relations. It included decision-making, but also the meaning, motivation and purpose behind these actions.
(ii) Resources, i.e. the medium through which agency is exercised. Access to resources affects capacity to make strategic choices.
(iii) Achievements, i.e. the outcomes of agency. Achievements refer to the extent to which the potential for people to live the lives they want is realized.
Kabeer emphasizes the transformative forms of agency and achievements that refer to a “greater ability of poor women to question, analyze and act on the structure of patriarchal constraint in their lives”.
This definition of empowerment has the characteristic of being encompassing and is complementary to the conceptual framework used in the analysis (see below). It is aligned with this study’s objective, which is to explore how women’s empowerment has been studied in relation to user fees, rather than to delineate it, to decompose it into components, or to measure it.

In the second step, all identified keywords were searched systematically in six health-focused databases: PubMed, CAB Abstracts, Embase, Medline, Global Health, and EBM Reviews (the latter four were accessed via the Ovid platform). Boolean logic operators (AND, OR) were used to combine terms, and a truncation sign (*) was added as an open-ended term (see Table 2). The search was conducted using only English terms, but language of results was extended to French and Spanish. The search was conducted on February 20, 2020, with no date limitation; all publications between 1946 and the search date were eligible. All articles were imported into EndNote, where duplicates were identified and removed.

**Table 2:** Search terms used for the scoping review

Boolean expression [all fields] (as run on Ovid and adjusted for other platforms)	
((women OR gender OR female*) AND (decision-making OR decision* OR empower* OR autonomy OR capabilit* OR bargaining OR self-determination) AND (“free health*” OR “user fee* removal” OR “removal of user fee*” OR “abolition of user fee*” OR “user fee* abolition” OR “user fee* exemption” OR “exemption of user fee*” OR “reduction of user fee*” OR “user fee* reduction” OR “healthcare subsid*” OR “health care subsid*” OR “obstetric care subsid*”))	

The third step took place after the complete screening of initial results (see screening process below). It was performed to identify potential additional papers by examining (i) the reference lists of all selected papers and (ii) their citations in Google Scholar. These new results were then screened by applying the same criteria as in the initial screening (see below).

### Eligibility criteria and screening process

**Fig. 1:**
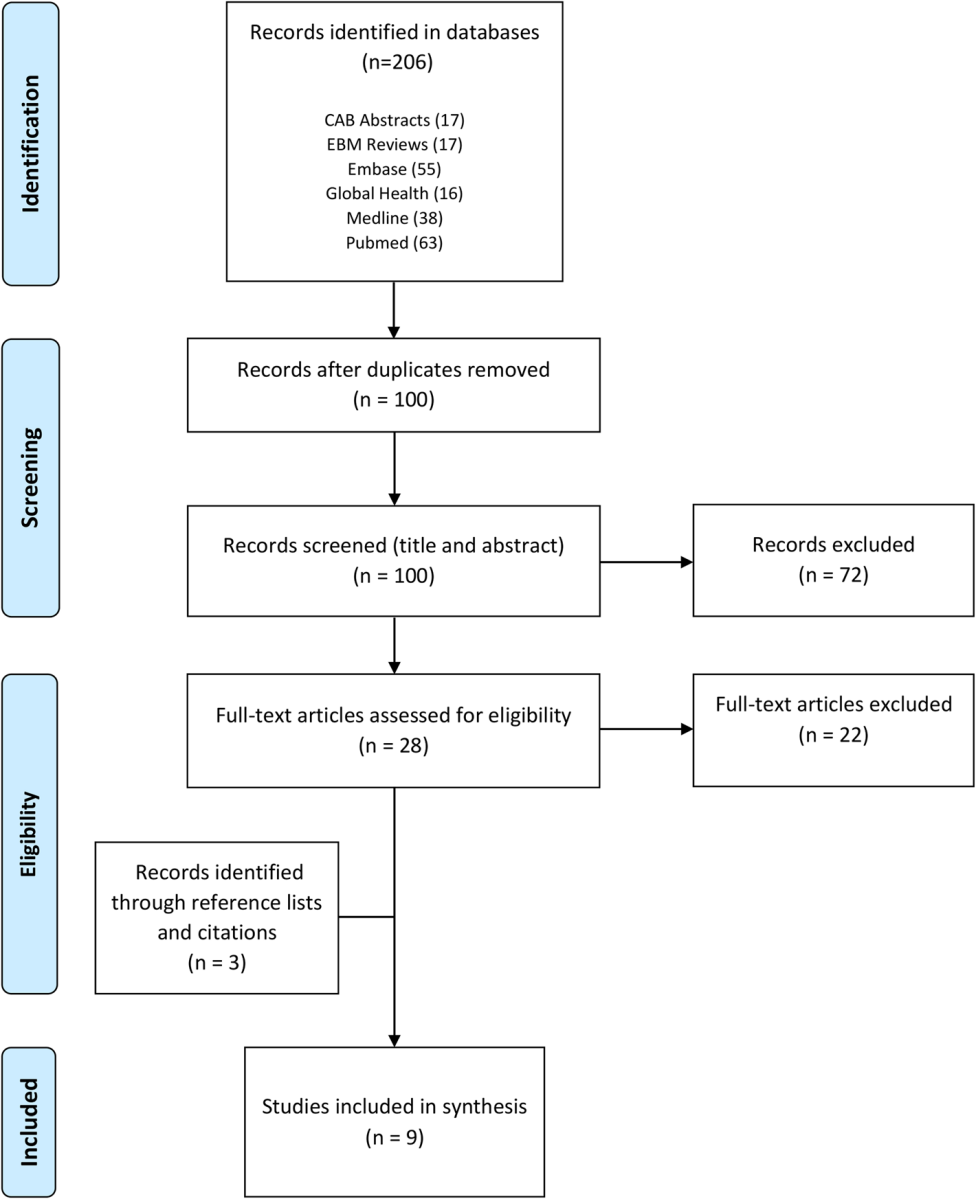
PRISMA flow chart with number of records at each step

Articles were screened in two stages. In the first stage, titles and abstracts were screened using three exclusion criteria (Table 3). Articles that met at least one exclusion criterion were removed. In the second stage, the remaining articles were read in their entirety and those presenting new evidence about the relationship between user fee policies and women’s empowerment were retained for the quality synthesis. Although there are some conceptual nuances between women’s empowerment and the other related search terms (such as autonomy in decision-making, women capability, agency, etc.), the former will be used throughout this review as an encompassing expression which, based on Kabeer’s definition (Table 1), denotes women’s abilities to pursue their objectives, to use resources and grasp opportunities, and to participate in decision-making [31, 32]. The screening process was conducted by two reviewers to minimize bias and enhance reliability. At the end of each step, reviewers compared results and reached agreement on which articles to include, with differences resolved through discussion with a third investigator. The search process and number of papers retained at each step is summarized in a PRISMA flow chart (Fig. 1).

**Table 3:** Inclusion and exclusion criteria

**Exclusion criteria (stage 1)**
• The title or abstract does not mention user fees paid by patients at healthcare facilities
• The title or abstract does not mention the concept of women’s empowerment
• The title or abstract does not mention a low-or-middle-income country (LMIC) as the study area
**Inclusion criteria (stage 2)**
• The article presents new evidence about the relation between presence/absence/change in user fees and women’s empowerment (or related term)

Since the focus was to provide a systematic scoping review, quality of the studies was not formally assessed against a grading scale; instead, as much information as possible is presented for the readers’ own critical assessment. Experimental, quasi-experimental and observational studies were all considered for inclusion.

### Analysis

**Fig. 2:**
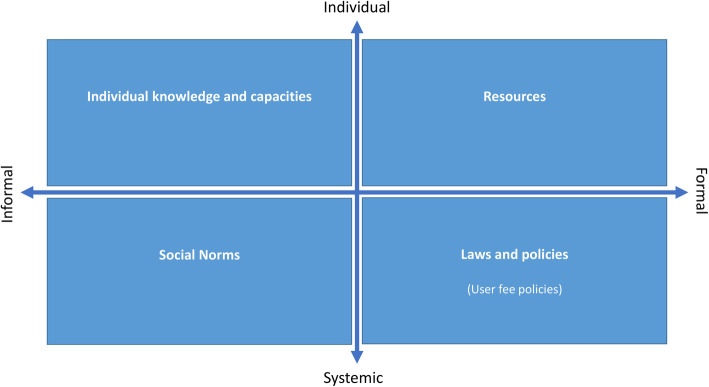
The Gender at Work analytical framework. Adapted from Rao A, Sandler J, Kelleher D. & Miller C (2016). Gender at work: theory and practice for twenty-first century organizations. London: Routledge

The Gender at Work framework was used to organize the material and to facilitate the presentation of the results. This framework was a priori deemed appropriate for a systematic scoping review on women’s empowerment, because it is multifactorial and holistic [33]. Very briefly, the Gender at Work framework contends that to be sustainable, change must occur at the individual, household, community and national levels simultaneously [34]. It is based on two dimensions: individual vs. systemic and formal vs. informal, and divided in four quadrants (Fig. 2). Applying this framework, user-fee removal policy was placed under the formal/systemic quadrant, while women’s empowerment was attributed to the individual/informal sphere. This analysis intends to highlight the interrelations between the quadrants, notably by exploring whether the introduction of a formal policy brings about changes in the other quadrants of the framework. Key themes in each quadrant were identified by one author (VP) and validated by a second (TD).

## Results

### Description of the material

Out of the 206 initial records, six papers met the inclusion and exclusion criteria. Three additional papers were identified by examining references and citations, for a total of nine papers included in the review [35–43]. The articles’ key characteristics are presented in Table 4. The nine papers described results obtained from six different studies: Samb et al.’s three papers [37, 42, 43] and Treacy et al.’s two papers [38, 39] are based on the same study. Studies were all conducted in SSA (Burkina Faso, Kenya, Mali, Sierra Leone), except one that was performed in India. Two of these countries are considered lower middle income (Kenya, India), while the rest are low-income countries. They all used qualitative methods for primary data collection. All articles have been published relatively recently (publication dates ranging 2012–2019). The corpus is also characterized by high variability regarding study objectives, scope of intervention, setting, and rhetoric or conceptual framework used in regard to women’s empowerment. Seven papers focus on countries where user fees were removed for most maternal health services, one concerns a country where user fees for maternal health remained (except for caesarean sections) and one covered a wider range of health-financing policies, including user fee removal.

**Table 4:** Main characteristics of the included studies

First author (year)	Country and setting	Objective	User fee policy in place	Type of study and participants	Main conclusion about the relation between user fee policies and women’s empowerment
Cornish et al. (2019) [41]	Sierra Leone	To explore the relationship between women’s economic empowerment and health decision-making	Free health care for pregnant women, breastfeeding mothers and children < 5	Qualitative	Indirect relation
	10 rural communities			In-depth interviews *N* = 29 women	Economic interventions support women’s economic empowerment, but women’s social empowerment, alongside economic empowerment, needs consideration
Johnson et al. (2012) [35]	Mali	To identify consequences of user fees on gender inequality, food insecurity and household decision-making	Fees are charged at point of care for consultations, diagnostic, medications and care services, excepted a few services that are free of charge (caesarean sections, HIV testing and treatment, malaria treatment)	Qualitative	Direct relation
	Peri-urban areas			Ethnographic life history interviews	User fees reduced agency for women in health care decision-making
				*N* = 24 women	
Kabia et al. (2018) [36]	Kenya	To explore how gender disability and poverty interact and influence how poor women in Kenya benefit from pro-poor financing policies that target them.	(i) Free maternity policy	Qualitative	Indirect relation
	County A (urban)		(ii) abolition of user fees in public primary healthcare facilities	In-depth interviews	Poor, disabled women continued to experience disempowerment within the health system despite free health care
	County B (rural)			*N* = 11 women with disabilities living in poverty	
			(iii) health insurance subsidy programme		
Samb et al. (2018)^a^ [37]	Burkina Faso	To examine the effect of free healthcare on women’s capability	Reproductive and maternal care (including emergency obstetric and neonatal services) are free of charge	Qualitative	Direct relation
	Rural areas in 3 health districts (Dori, Sebba, Ouargaye)			Semi-structured interviews, documentary analysis and non-participant observation	Free healthcare contributed to strengthen women’s capability related to healthcare
				*N* = 64 (40 women, 16 members of a health center management committee and 8 healthcare workers)	
Samb et al. (2015)^a^ [43]	Burkina Faso	To examine the effect of free healthcare on women’s capability	Reproductive and maternal care (including emergency obstetric and neonatal services) are free of charge	Qualitative	Direct relation
	Rural areas in 3 health districts (Dori, Sebba, Ouargaye)			Semi-structured interviews, documentary analysis and non-participant observation	Free healthcare contributed to strengthen women’s capability related to healthcare
				*N* = 64 (40 women, 16 members of a health center management committee and 8 healthcare workers)	
Samb et al. (2013)^a^ [42]	Burkina Faso	To examine the effect of free healthcare on women’s capability	Reproductive and maternal care (including emergency obstetric and neonatal services) are free of charge	Qualitative	Direct relation
	Rural areas in 3 health districts (Dori, Sebba, Djibo)			Semi-structured interviews	Free healthcare contributed to strengthen women’s capability related to healthcare
				N unknown	
Treacy et al. (2015)^b^ [38]	Sierra Leone	To explore the perceptions and decision-making processes of women related to childbirth in a context of free healthcare.	Free health care for pregnant women, breastfeeding mothers and children < 5	Qualitative	Indirect relation
	2 villages in the Northern Province			Focus groups and in-depth interviews *N* = 71 (44 women and 27 men)	Decision-making processes during childbirth are dynamic, intricate and need to be understood within broader social context. For this reason, free healthcare initiatives have limited impact.
Treacy et al. (2018)^b^ [39]	Sierra Leone	To explore who and what influences the decisions made by women, and how the position of women in society impact upon these processes in a context of free healthcare	Free health care for pregnant women, breastfeeding mothers and children < 5	Qualitative	Indirect relation
	2 villages in the Northern Province			Focus groups and in-depth interviews	Gender inequities remain despite the introduction of free health care.
				*N* = 61 (number of women unknown)	Decisions during childbirth are influenced by constraints of poverty and other social determinants (unequal power, social norms, etc.)
Witter et al. (2017) [40]	India (secondary data analysis)	To explore which financing reforms are likely to be the most effective at accelerating progress toward universal health coverage while at the same time addressing gender inequities	Different health-financing policies including user fees, health insurance, vouchers and conditional cash transfers	Rapid review of health literature and case study	Indirect relation
					Public financing mechanisms such as user fee abolition are not sufficient to reduce gender inequities because they do no tackle the social determinants that undermine access to healthcare

^a^ and ^b^ indicate papers derived from a same study

Only two studies, representing four papers [35, 37, 42, 43], have identified and examined a direct association between user fee policies and women’s empowerment. The remaining five articles have addressed this issue indirectly, mostly by examining gender equality or women’s decision-making in the context of free healthcare.

### User fee policies and their direct influence on Women’s empowerment

Two studies identified such a direct association. The first was conducted in a region in Burkina Faso where reproductive healthcare was completely free of charge for women [37, 42, 43]. It argues that user fee removal for obstetric care has a transformative effect on women through three different mechanisms. First, it improves women’s capability to make health decisions, since they no longer have to negotiate access to household resources prior to receiving healthcare. Second, it increases their self-esteem and preserves their dignity by removing the burden of having to sell goods or borrow money from neighbours or family, especially their husbands. Third, their social position and bargaining power within the household increases due to greater decision-making and reduced marital tension, a phenomenon also observed indirectly in another of the studies under review [41]. Despite these benefits, the study suggests that user fee abolition policies did not change practices deeply rooted in the social or cultural structures, particularly regarding contraception. However, it was observed that some women did start using contraception once it was free, mostly without their husbands’ approval. As argued by the authors, this is illustrative of the transformative capacity of this policy, even if it was limited [37].

The second study was conducted in a context where women had to pay for most healthcare services [35]. Results suggest that the presence of user fees reinforces pre-existing gender inequalities and undermines women’s agency to make healthcare-related decisions. The presence of user fees renders women more dependent on their husbands for decision-making related to healthcare, both for themselves and for their children. The husbands decide based on a “medical-financial diagnosis” that they perform and upon which they allocate financial resources. In addition, costs for medication and related health services sometimes reduce the amount of money that women can spend to buy food. This situation not only raises food security issues in their household, but can also lead to women’s stigmatization and social isolation, especially if their children are undernourished.

### Free healthcare, but remaining issues of resources

Control over resources was reported by all studies as a key issue for women’s decision-making related to healthcare, even in a context where it was officially free of charge. Economic resources, education and information were the three most common types of resources that reportedly influenced women’s empowerment.

#### Economic resources

Five papers highlighted the fact that, despite user fee removal, other barriers related to costs limit access to healthcare services. These include transportation costs, lost work time, and costs of medications and other services not covered by the policy [35]. These costs concerned not only the patient, but also the accompanying person. In addition, informal payments were sometimes still required at the health center, either due to abusive requests made by healthcare providers or because patients wanted to ensure good treatments and/or show appreciation [37, 39]. Lastly, some individuals chose to avoid free healthcare and preferred to continue paying user fees, because of perceptions that payment is an indicator of higher quality services [41].

The magnitude of these remaining costs varied from one context to another, but they remain significant for women’s empowerment. Indeed, in contexts where women have limited access to economic resources, their autonomy in healthcare decision-making remains limited. In some cases, the issue is not the absence of economic resources, but control over them [43]. When the husband controls the household finances and the family must decide whether to bring a sick child to a health facility, his judgment prevails, even if the wife is the primary caregiver [41]. When women earn their own income over which they have control, they have more autonomy to make healthcare decisions [39].

#### Access to information

Information about healthcare was described as a resource that affects women’s decision-making. Removal of user fees does not intend to increase women’s knowledge about health or healthcare services per se. In fact, two studies showed that, despite free healthcare, women’s empowerment was limited because they lacked information about healthcare services, which in turn potentially further undermined their confidence to interact with healthcare providers [36, 40]. Poor women with disabilities were particularly vulnerable and disempowered in this regard [36].

In the same vein, a study conducted in Sierra Leone highlighted the secrecy surrounding childbirth, i.e., the fact that information about pregnancy the childbirth process was restricted to those who had experienced it [38]. This lack of access to information affected primigravidae women’s ability to make and justify choices during pregnancy and labour. Even if user fees were removed in health facilities, these women were heavily dependent on advice and decisions made by others, specifically their mothers (or mothers-in-law) and traditional birth attendants from the community.

#### Access to education

A more distal factor of women’s health-related autonomy is education, conceptualized here as a resource that affects access to remunerated positions, control over monetary resources, access to information about healthcare, and literacy in health. As documented by one study, the fact that fewer women finish secondary school education or have public or remunerated positions compared to men negatively affects their ability to negotiate and participate in household decision-making related to healthcare, even in the absence of formal user fee [39]. Another study highlighted the fact that women with disabilities are particularly vulnerable; they tend to be less educated compared to men and women without disabilities, which further limits their agency and ability to receive (free) healthcare services [36].

### Free healthcare, but persisting influence of social norms

Although social norms are intangible, they are key determinants of women’s empowerment, as observed in most of the papers under review (8/9). Three dimensions of social norms were identified during the analysis of the corpus: (i) gender-based division of labour within the household, (ii) community beliefs surrounding reproductive health, and (iii) patient-healthcare provider relationship.

#### Gender-based roles in the household

Most of the studies (5 out of 7) showed that constraints related to gender roles within the household limit women’s autonomy and empowerment. Men are usually considered as having the role of provider, head of the household, and decision maker. This patriarchal system, as highlighted in the studies conducted in Sierra Leone and Burkina Faso, gives husband control over decisions relating to healthcare [39, 41, 42]. It imposes itself not only over their wives, who need the husband’s permission before seeking care or receiving treatment, but also over health personnel, who can be reluctant to perform some medical procedures without the husband’s permission [37, 39].

Women’s household and childcare responsibilities limit her access to healthcare services, even if they are free. Studies show that, even if they are willing to seek care, women must first find someone to take care of their children and other domestic responsibilities during their absence [36, 39]. This type of opportunity cost represents a barrier to healthcare access for most women, since they regularly engage in formal or informal economic activities and provide, partially or totally, for their household.

#### Community-based practices surrounding reproductive health

Nearly all studies mentioned that reproductive health is associated to (or, to some extent, shaped by) community-based practices, beliefs and traditions. Even in the context of free healthcare, these structural factors limit women’s autonomy in decision-making. Because of these factors, women continue to refrain themselves from changing place to deliver or from avoiding traditional birth attendants, as illustrated in the study conducted in Sierra Leone [38]. Use of contraception is another example identified in the literature of women’s limited autonomy due to social norms and cultural pressure; even when it was free of charge in Burkina Faso, its use remained very limited and depreciated [37, 42]. Finally, in some areas, community distrust or suspicion about anything that is free is common [41]. In such contexts, women can be discouraged from using free healthcare services.

Finally, health-care decision-making was portrayed as a complex, collective process. As discussed in one study, in some contexts, it does not rely solely on one individual (the husband) but rather involves several members of the household or the community [38]. Older spouses or mothers-in-law within the household, as well as older women or traditional birth attendants within the community, may influence decisions surrounding childbirth. Therefore, women have to socially negotiate decisions about their health and conform to community expectations, in addition to negotiating for resources controlled by their husbands.

#### Patient–healthcare provider relationship

Social norms, embodied and reproduced by healthcare providers, also contributed to limit women’s empowerment. Disrespectful and discriminatory attitudes and behaviors experienced by women in health facilities were reported by several studies as disempowering, in addition to discouraging them from seeking healthcare in the future [36, 39, 40]. As for most patients, the relationship between women and healthcare providers is characterized by power imbalance. However, because of gendered norms, women are more prone to be exposed to disrespect and humiliation practices from healthcare professionals, and even in some cases to verbal and physical violence, extortion, and abuse. Despite free healthcare, these negative attitudes contribute to stigmatizing women, to reduce their satisfaction and use of services at the health facilities, and to limit their autonomy in decision-making. Similarly to what was observed above, poor women or women with a disability or less education are particularly vulnerable to these forms of prejudice, notably because they required extra assistance from healthcare providers [36].

### Recommendations

Several studies expressed recommendations to better address gender barriers and issues related to user fees [36–40]. The main recommendations are summarized in Table 5.

**Table 5:** Recommendations to better address gender issues related to user fees

Main recommendations expressed in the included studies
• Adopt a multi-sectoral approach that goes beyond user fee policies [35, 36, 39]
• Increase collaboration between different departments (i.e., Health, Agriculture, Education, Economic Development, etc.) [37, 39]
• Address the structural determinants of gender inequalities in health [36, 39]
• Increase women’s access to monetary resources and education [36, 39]
• Adapt intervention to the social context and local conceptions of gender inequality [35–38]
• Use an intersectional lens when planning interventions due to the multidimensional nature of vulnerability and inequity [35, 36, 38, 39]
• Consider user fee abolition not only for reproductive health, but for women’s health in general [36, 39]
• Involve men in reproductive health planning and implementation [37]
• Increase health governance, transparency and accountability [35, 39]

## Discussion

This review explored the relations between user fee policies and women’s empowerment. The evidence suggests that user fee removal can contribute, in some contexts, to improving women’s capability to make health decisions and that, reciprocally, the presence of user fees reduces women’s agency in healthcare decision-making and reinforces gender inequalities. User fee abolition does not give additional resources —or control over them— to pregnant women, but reduces their need to negotiate within the household for obtaining money. Intra-household bargaining dynamics have been identified in the literature as a common barrier to healthcare access in low-income countries [44–46]. Even if it does little to challenge it, removing user fees can compensate for women’s low bargaining power [47], and increase their agency to receive healthcare for themselves or their children. However, findings reveal that, even in a context of free healthcare, some non-financial and even financial costs persist. Several studies have examined the out-of-pocket payments that remain even when user fees are officially abolished —these costs are not negligible and continue to represent a barrier to healthcare access [48–51]. Arguably, the need for women to negotiate over economic resources is reduced, but still present.

Removal of user fees does not give women better access to economic resources, contrary to microfinance or cash transfer programs. These programs do, in some instances, contribute to increasing women’s empowerment [25]. That being said, it is important to distinguish between access to and control over economic resources when considering women’s empowerment [52–54]. Studies have shown that an intervention might lead to better access to economic resources for women, but that its impact on women’s empowerment is limited as long as women do not have decisional autonomy over them [55–57]. In some instances, microfinance programs were shown to have disempowering effects when women had little or no control over micro-credits [58, 59]. Arguably, while access and control over economic resources are relevant to empower women, other factors must be taken into consideration.

This stresses key elements identified in the literature review, i.e., the structural influence of gendered norms that, despite free healthcare, continue to limit women’s autonomy. To the best of our knowledge, none of the user fees abolition policies specifically include interventions to influence social norms. It has been demonstrated that women continue to ask their husband’s (or someone else’s) permission to visit a health center, even if they have the means to pay for it [60]. This review suggests that gendered norms are particularly important in defining women’s practices regarding reproductive care and childbirth, even if they are provided free of charge at health facilities. This is aligned with a large body of evidence in the feminist literature that has already underlined how reproduction, in very different contexts, is governed by social norms of high magnitude [61, 62]. User fees abolition policies have the potential to lead to unintended negative consequences if they disregard these norms —similarly to what was observed in some micro-credit and vocational training programs, which can put women at higher risk of domestic violence because they challenge prevalent social norms [63, 64]. The parallel is preoccupying since one of the studies under review related that some women decided to use contraception —once it was available free of charge— without their husband’s approval or knowledge [37]. Considering the norms, historical coercion and structural violence surrounding family planning, it seems important to complement user fee abolition with measures to promote gender equality and mitigate risk of domestic violence [65].

Another key normative mechanism relates to women’s disempowering experiences in their interactions with the healthcare system. Several of the studies under review revealed that women commonly endure discriminatory practices, disrespectful attitudes, violence and stigmatization from healthcare providers. The quality of the provider-patient relationship has recently been acknowledged as a major issue in LMICs, for healthcare access but also for women’s rights [66, 67]. If not addressed in its interventional framework, a user fee abolition policy can potentially contribute to increasing women’s disempowerment through two mechanisms. First, it intensifies the average number of contacts —and, therefore, the number of disempowering experiences— between women and healthcare providers [16, 19]. Second, it can aggravate the patient-provider relationship because of the health personnel’s dissatisfaction with the increased workload, or its suspicion that community members overuse healthcare services [68–70]. User fee abolition policies need to promote respectful maternity care as a basic human right, which entails not only health systems strengthening, but also healthcare educational training to specifically combat negative attitudes and behaviors [71].

Intersectionality was a transversal theme in this review. It showed that women with disabilities, with less education or living in poorer households face additional challenges in benefitting from free healthcare policies [72]. Even in a context of free healthcare, these groups need more resources (money, information, disability-friendly transportation and facilities, etc.) to access healthcare services, and they are more prone to experience disrespect and abuse, rooted in prejudicial social norms. There has been a debate whether or not all socioeconomic groups equally benefit from user fee abolition [19, 73, 74]. This inconclusive finding might come from the intersection of poverty with other types of social vulnerabilities or disabilities in the production of effects. In other contexts, it was found that women who are HIV-positive, unwed or engage in sex work are particularly at risk of facing discrimination and stigma in health facilities [66, 67]. Gender interacts with other social dimensions, such as class, race, age, sexual orientation, educational level, marital status, and are powerful determinants of women’s experiences within the healthcare system.

### Theoretical considerations (gender at work framework)

The Gender at Work framework was useful in categorizing results into different structural dimensions and highlighting their interrelations [33]. The four dimensions should not be conceived as totally separate; the categorical representation was useful to organize results and to analyze the interactions between factors of different nature. In addition, by presenting a multi-level, macroscopic gender analysis, this framework does not intend to decompose the concept of women’s empowerment itself. Its holistic perspective was deemed appropriate for the purpose of this review because the aim was to study the impact of a formal, systemic intervention on women’s empowerment [31, 75].

A key finding of this review is that user fee removal (introduction of a formal, systemic policy), on its own, may not be enough to significantly improve women’s empowerment (informal, individual capacities), although it can contribute to it. Complementary strategies should focus on two other dimensions by challenging restrictive gender norms and improving women’s access to different types of resources (money, information, and education). Indeed, user fee abolition policies may partially reduce women’s needs for financial resources, but their effects on individual beliefs and capacities, as well as on restrictive social norms are limited. As Rao et al. explain, “As important as resources are, [ …] increased resources may have limited impact on women’s capacity to change or challenge institutional norms regarding their position in society” [33].

In the same vein, several of the studies under review recommend measures that go beyond financial and health-specific considerations – e.g. initiatives that support education for girls and campaigns that challenge harmful cultural norms (see Table 5). The use of a gender-based lens reveals that healthcare access should no longer be reduced to a formal, sectorial policy (user fees exemption, cash transfer, insurance scheme, etc.), but should include measures to strengthen women’s overall position in society and address gender inequalities at a systemic level. On the road to universal health coverage, multi-sectoral approaches are needed to promote sustainable gender equality and achieve better health outcomes for women [76]. This entails an in-depth understanding of the complexity of intra-household decision-making processes and their interactions with the local normative context in which individuals are embedded. This also includes taking into account how the concepts of ‘autonomy’ and ‘empowerment’ may be interpreted differently according to socio-cultural contexts (for example in contexts where collective values supersede individual values) [77, 78].

### User fees abolition, Women’s empowerment and access to healthcare

This review of qualitative studies indicates that the relationships between user fees abolition, women’s empowerment and access to healthcare are manifold. As discussed above, the articles suggest that user fee policies have the potential to directly influence —positively and negatively— women’s empowerment. Interestingly, as recently highlighted in two systematic reviews, decision-making autonomy in women is positively associated with healthcare utilization in SSA countries [79, 80]. Therefore, women’s empowerment is likely to act as a mediator between user fee abolition and access to healthcare. At the same time, there is a vast literature showing that user fees abolition policies have a direct impact on the utilization of maternal healthcare services [13, 19]. However, the evidence gathered here shows that women’s empowerment can modulate this impact —women with more autonomy in decision-making will benefit more from user fee removal. Thus, empowerment can also be interpreted as an effect modifier [81].

The role of women’s empowerment has been overlooked in the impact evaluation studies of user fees abolition [82]. Indeed, despite the number of studies that quantitatively have assessed the effects of user fee abolition policies on healthcare utilization, it is surprising that the influence of women empowerment as a mediator or modifier remains to be empirically validated [81, 83]. Arguably, this is an important factor that needs to be taken into consideration in the impact assessment, along with the other common variables (age, sex, socioeconomic status, remoteness of the household, etc.). As argued elsewhere, women’s empowerment and gender equality are essential stepping stones to achieving universal health coverage [84, 85]. While the SDGs acknowledged that gender equality must become a prism for action, the results gathered here suggest that it is not sufficiently taken into consideration in the studies examining one of the most popular interventions to achieve universal health coverage. Arguably, this issue is disregarded not only in research, but also in policy-making, in program planning and monitoring, and in process evaluation [2].

### Limitations

This scoping review is subject to some limitations. There was a surprisingly small number of papers that met the inclusion criteria. Several measures were taken to face this challenge, including a careful review of the research terms and exclusion criteria, an extension of the search date, as well as including a final search based on references and citations of included papers. Despite this, only nine papers could be included in the review and, among these, five were partly redundant because they analyzed the same material. Geographical distribution is also limited, since all studies but one were conducted in SSA, and they all concern countries with low- to lower-medium income. In addition, all the included studies qualitatively explored women empowerment issues in conjunction with user fees policies. Results about the associations between user fees policies, women’s empowerment and access to healthcare are to be used with caution and in the perspective of generating hypotheses or guiding future research, rather than in a causal framework. Also, there are numerous definitions of women’s empowerment, and the literature has highlighted the challenges and complexities related to its measurement [31, 86]. This review did not attempt to distinguish between ‘empowerment’, ‘autonomy’ and ‘agency’ and, as such, relied upon an encompassing concept. Finally, while the screening process was conducted by two researchers to increase validity, the qualitative analysis phase was conducted by one author. Since the focus was to provide a scoping review, quality of the studies was not formally assessed against a grading scale; instead, as much information as possible is presented for the readers’ own critical assessment.

## Conclusion

This systematic scoping review suggests that user fee abolition policies may remove the need for women to bargain for economic resources to access healthcare services. However, on their own, these policies are not enough to improve women’s empowerment or, more specifically, their autonomy in healthcare decision-making. Indeed, these policies do not contribute to improving women’s control over resources or challenging prejudicial social norms, which are key actions to achieve greater gender equality [87]. While a growing number of LMICs are abolishing user fees in health facilities to increase access to healthcare, there is an opportunity to adopt multi-sectorial, equitable approaches that aim to increase women’s empowerment in the process. In addition to its intrinsic value as an essential human right, gender equality is an indispensable stepping stone to improve utilization of healthcare services. For these reasons, it might be appropriate to redefine the focus from “access to healthcare” to “gender equitable access to healthcare” in the planning, implementation and assessment of universal health coverage interventions [84].

## Data Availability

All studies under review are published and can be accessed on the website of the editing journals.

## Abbreviations

LMICs: Low- and middle-income countries
MDGs: Millennium Development Goals
SDGs: Sustainable Development Goals
SSA: Sub-Saharan Africa
SSR: Systematic scoping review
UHC: Universal health coverage
